# Design, Analysis, and Reporting of Crossover Trials for Inclusion in a Meta-Analysis

**DOI:** 10.1371/journal.pone.0133023

**Published:** 2015-08-18

**Authors:** Tianjing Li, Tsung Yu, Barbara S. Hawkins, Kay Dickersin

**Affiliations:** Center for Clinical Trials and Evidence Synthesis, Department of Epidemiology, Johns Hopkins Bloomberg School of Public Health, Baltimore, Maryland, United States of America; University of Chieti, ITALY

## Abstract

Randomized crossover trials are clinical experiments in which participants are assigned randomly to a sequence of treatments and each participant serves as his/her own control in estimating treatment effect. We need a better understanding of the validity of their results to enable recommendations as to which crossover trials can be included in meta-analysis and for development of reporting guidelines.

**Objective:**

To evaluate the characteristics of the design, analysis, and reporting of crossover trials for inclusion in a meta-analysis of treatment for primary open-angle glaucoma and to provide empirical evidence to inform the development of tools to assess the validity of the results from crossover trials and reporting guidelines.

**Methods:**

We searched MEDLINE, EMBASE, and Cochrane’s CENTRAL register for randomized crossover trials for a systematic review and network meta-analysis we are conducting. Two individuals independently screened the search results for eligibility and abstracted data from each included report.

**Results:**

We identified 83 crossover trials eligible for inclusion. Issues affecting the risk of bias in crossover trials, such as carryover, period effects and missing data, were often ignored. Some trials failed to accommodate the within-individual differences in the analysis. For a large proportion of the trials, the authors tabulated the results as if they arose from a parallel design. Precision estimates properly accounting for the paired nature of the design were often unavailable from the study reports; consequently, to include trial findings in a meta-analysis would require further manipulation and assumptions.

**Conclusions:**

The high proportion of poorly reported analyses and results has the potential to affect whether crossover data should or can be included in a meta-analysis. There is pressing need for reporting guidelines for crossover trials.

## Introduction

**Fig 1:**
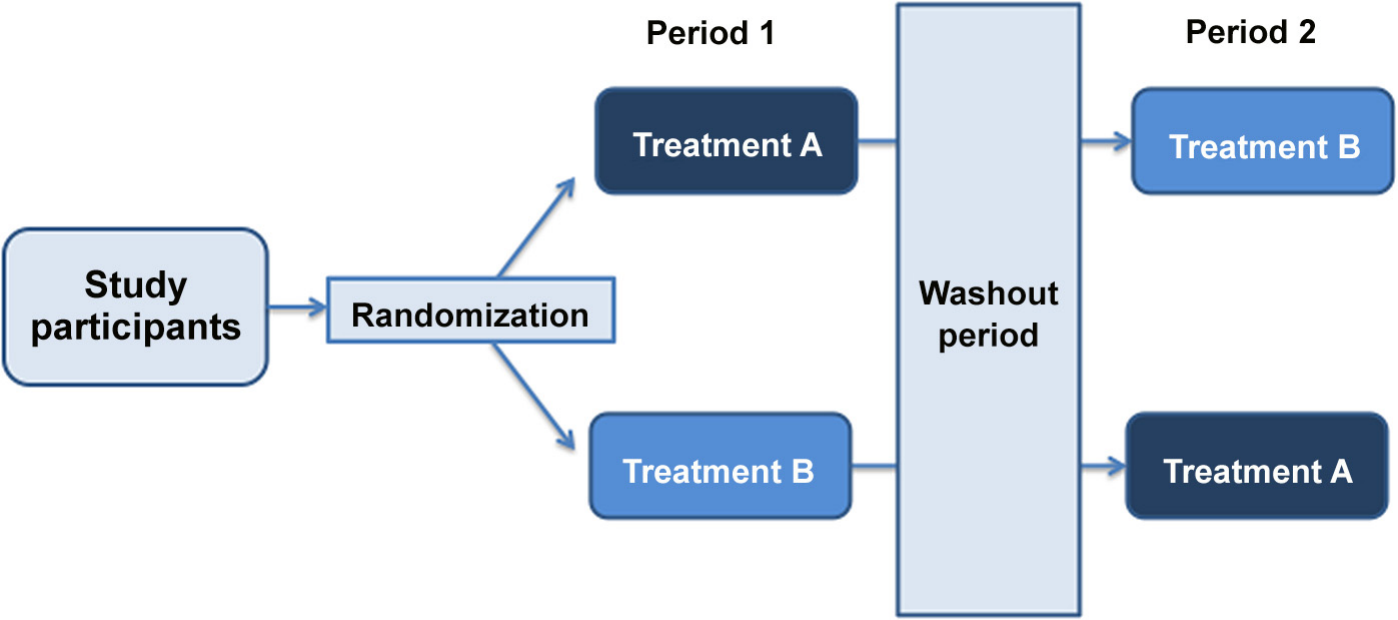
Illustration of the design and analysis of a crossover trial. *Carryover effect*: If A is an active intervention and B is a placebo, then the BA sequence is unlikely to be affected by a carryover effect, but the AB sequence is potentially susceptible. In the AB sequence, when some effect of the active intervention A is carried over to the second period, placebo could demonstrate artificial “effectiveness”. Under this scenario, the treatment effect of A compared to B would be under-estimated for the AB sequence, and so for both sequences combined [7]. Thus, if there are differential carryover effects in the two treatment sequences, the design can yield biased estimates of the treatment effect [1–4]. *Washout period*: To minimize a possible carryover effect between periods in a crossover trial, investigators use a “washout” phase that is sufficiently long to eliminate the first intervention’s effects [1, 2]. Although some researchers have recommended estimating and testing for the carryover effect, and when the effect is present, analyzing data collected from the first period only, this method has been shown to lead to biased estimates of effect [9]. Senn and others have taken the position that the crossover design should be used only when the assumption that there is a minimal carryover effect is likely to hold [1]. In such cases, instead of testing for carryover effect, one proceeds as if there were none. There also is the ethical consideration with using a washout period in participants with a chronic condition; in such cases, giving no treatment may not be in a participant’s best interests.

Randomized crossover trials are clinical experiments in which participants are assigned randomly to a sequence of treatments and each participant serves as his/her own control in estimating treatment effect [1,2]. For example, in an AB/BA design, the simplest form of a randomized crossover trial, participants are assigned randomly to either treatment A followed by treatment B, or treatment B followed by treatment A (Fig 1). Because both treatments are evaluated for the same individual, the treatment effect can be estimated based on an average of within-individual differences (Fig 1, Tables 1 and 2) [1–3]. Given this property, a crossover trial can theoretically achieve the same precision as a parallel group trial with only half the sample size. The required sample size is reduced further because outcomes measured in the same individual generally have a smaller variance than outcomes measured between individuals [1,2].

**Table 1:** Analysis of a crossover trial–an illustrative example.

	Treatment period		Within- individual difference (A–B)
	1	2	
Participants	Measurements		
**A then B**			
1	30	20	10
2	32	19	13
3	28	20	8
4	32	24	8
5	31	22	9
**B then A**		** **	** **
6	22	30	8
7	23	29	6
8	20	31	11
9	25	32	7
10	21	28	7

The mean and standard deviation of the ***within individual difference*** captures the treatment effect and the paired nature of the design; and should be used as the basis for constructing confidence intervals and a hypothesis test. In this example, assuming that there is no carryover or period effect, and that the within individual differences are approximately normally distributed, the estimated treatment effect is 8.7 and the standard deviation is 2.1 (standard error = 0.67). The 95% confidence interval ranges from 7.2 to 10.2 (see results below).

**Table 2:** Results of the illustrative crossover trial presented in Table 1.

	Treatment period		
Treatment sequence	1	2	Within-individual difference: A-B
**A then B**			
Mean (SD)	30.6 (1.67)	21 (2.00)	9.6 (2.07)
Sample size	5	5	5
**B then A**			
Mean (SD)	22.2 (1.92)	30 (1.58)	7.8 (1.92)
Sample size	5	5	5
**Treatment effect**			
Mean (SD)	-	-	8.7 (2.11)
95% confidence interval			7.2 to 10.2
Sample size	-	-	10
T-test for paired samples	-	-	<0.001

Several aspects of crossover trial design are critical to the potential risk of bias in the findings and interpretation. The first design consideration is that treatment from one period may have a residual effect that persists into the subsequent period, particularly when there is no “washout” between periods [1,2]. This is called a *carryover effect* (Fig 1). The second consideration is a *period effect*, which can occur when the treatment effect is not constant over time resulting in treatment by period interaction [1,2]. Period effect is more likely to occur when the treatment periods are long and when the underlying medical condition is not stable. Third, dropouts and missing data usually have a larger impact on crossover trials than on parallel group trials because missing data from one period preclude the within-individual comparison for all who enrolled in the trial [4]. Finally, there are situations in which the crossover design is inappropriate; for example, when the treatment in an earlier period (e.g., a vaccine) permanently alters the course of the condition such that, on entry to the next period, the participant characteristics systematically differ from their initial state [1–3].

The analysis and reporting of crossover trials should also account for the paired nature of the design [3] (Tables 1 and 2). This means that the treatment effect and associated precision are calculated based on within-individual treatment comparisons so that the potential gains in precision and statistical efficiency by choosing a crossover design are realized.

We became interested in the problem of crossover trials in the context of a systematic review and network meta-analysis we undertook, which identified a large number of trials that used a crossover design. We did not want to eliminate them from our analysis, as this would mean wasted information. On the other hand, we were faced with the challenge of deciding which of the trials should be included in the network meta-analysis and how. Our objective was to evaluate the design, analysis, and reporting characteristics of these crossover trials and provide empirical evidence to inform the development of tools to assess the validity of the results from crossover trials and reporting guidelines for crossover trials.

## Methods

### Selection of studies

We examined randomized crossover trials eligible for a systematic review and network meta-analysis that we are conducting on the comparative effectiveness of medical interventions for ocular hypertension and open-angle glaucoma. The main inclusion criteria of the systematic review were: randomized controlled trials (RCTs) that assigned to each treatment ≥10 participants of any age or gender with physician-diagnosed ocular hypertension or primary open-angle glaucoma; and trials comparing at least one medical intervention with no treatment/placebo or another medical intervention. We set no maximum or minimum limit on the duration of treatment; however, we included only trials in which participants had been followed for ≥28 days after randomization.

We searched the Cochrane Register of Controlled Trials (CENTRAL) in *The Cochrane Library*, MEDLINE, and EMBASE on November 17, 2009 following a search strategy that was published previously [5]. Two individuals independently assessed titles and abstracts and then full text articles to identify eligible RCTs. Two individuals working independently identified the crossover trials within the total group for this study. We resolved disagreements between the two reviewers through discussion or consultation with a third person.

### Data collection and analysis

For each included crossover trial, two individuals (at least one with statistical expertise) independently abstracted data using an electronic data collection form developed, pilot-tested, and maintained in the Systematic Review Data Repository (S1 File), adapting some data items from a previous study of crossover trials [6]. We resolved disagreements between the two reviewers through discussion or consultation with a third person.

For each trial report, we recorded the rationale provided by the authors for using a crossover design, information on number of interventions being compared, sample size calculation, statistical analysis methods stated in the methods section of a report, and whether a washout period was used. We reviewed whether carryover and period effects were mentioned anywhere in the report and how the two effects were addressed in the data analysis of treatment effects. When change from baseline was reported as an outcome metric, we abstracted information on which baseline was used for calculating change (i.e., “before the start of the first treatment” or “after the completion of one treatment and before the start of the next treatment”). We also abstracted information on whether and how the investigator dealt with missing data and how results were reported. We assessed whether it was possible to calculate precision of effect that accounts for the paired nature of the design when not reported by the investigators, so that the study could be included in a meta-analysis.

We tabulated the number and proportion of trials reporting each of these characteristics. All analyses were conducted using STATA 13 (StataCorp. 2013. *Stata Statistical Software*: *Release 13*. College Station, TX: StataCorp LP.).

## Results

We identified 83 crossover trials (82 publications) eligible for inclusion in our systematic review; these trials constitute 16% of all eligible studies for our planned systematic review and network meta-analysis within this time period.

### Reporting of design

In terms of design characteristics, only a small fraction of the crossover trial investigators (5%, 4/83) provided a rationale for using a crossover design (Table 3). A large majority of the trials (88%, 73/83) examined two treatments. A pre-planned sample size calculation was reported for about half of the trials (54%, 45/83). Fewer than one half (41%, 34/83) reported using a washout period before the next treatment was started; a further 16% (13/83) stated why a washout period was not needed.

**Table 3:** Reported design characteristics of included crossover trials (n = 83).

Study characteristics	Frequency	
	Number	%
**Provided a rationale for using a crossover design**	4	5
**Number of treatments compared**		
Two^1^	73	88
Three	9	11
More than three	1	1
**Used a washout period before switching to the next treatment**		
Yes	34	41
No, reason(s) stated	13	16
No, no reason stated	35	42
Cannot tell	1	1
**Sample size and power** ^**2**^		
Pre-planned sample size calculation	45	54
*Post hoc* power calculation	14	17
Not reported	25	30

^1^Seventy-two of 73 trials used an AB/BA design, in which participants are randomly assigned to one of two sequences: treatment A followed by treatment B or treatment B followed by treatment A.

^2^One trial reported both a pre-planned sample size calculation and a *post hoc* power calculation.

### Reporting of analysis

The methods used for data analysis are of critical importance for those interpreting the findings (Table 4). Almost all trials (99%, 82/83) used data from more than one treatment period to estimate the treatment effect. However, only three- quarters of the trials (76%, 63/83) stated that the analysis accounted for the crossover nature of the design, that is, that each participant served as his or her own control. Ten percent (8/83), 17% (14/83), and 14% (12/83) of trials mentioned testing for the presence of carryover effect, attempted to deal with it in the analysis, or commented on it in the discussion section, respectively. Similarly, 18% (15/83), 23% (19/83), and 10% (8/83) of trial reports mentioned testing for the presence of period effect, attempted to deal with it in the analysis, and discussed it, respectively.

**Table 4:** Reported analysis characteristics of included crossover trials (n = 83).

Study characteristics	Frequency	
	Number	%
**Data used for analysis**		
From more than one period	82	99
Cannot tell	1	1
**Accounted for paired data nature of crossover trials in calculating treatment effect** ^**1**^		
Yes	63	76
No	7	8
Cannot tell	13	16
**Carryover effect**		
Tested for presence of carryover effect	8	10
Attempted to deal with carryover effect in the analysis	14	17
Discussed carryover effect	12	14
**Period effect**		
Tested for presence of period effect	15	18
Attempted to deal with period effect in the analysis	19	23
Discussed period effect	8	10
**Baseline values used for calculating “a change score from baseline” (n = 54)** ^**2**^		
Values obtained before the start of the first treatment	30	56
Values obtained after the completion of the first treatment and before the start of the second treatment	14	26
Both	1	2
Cannot tell	9	17
**Methods for handling missing data (n = 62)** ^**3**^		
Complete case analysis	52	84
Statistical methods for handling missing data	1	2
Not reported/cannot tell	9	15

^1^Based on the judgment of the data abstractor.

^2^Fifty-four trials reported a change score from baseline as an outcome measure.

^3^Sixty-two trials reported having some missing data; the remaining 21 trials did not report having any missing data.

Of the 54 trials that reported analyzing change in intraocular pressure from baseline, half of them (56%, 30/54) used the value measured before the start of the first treatment for calculating the change; a quarter of them (26%, 14/54) used the value measured after the completion of one treatment but before the start of the next treatment; one trial used both (2%); and the remaining trials (17%, 9/54) did not report clearly what had been used as the baseline value. Of the 62 trials with missing data, a large proportion (84%, 52/62) used complete case analysis (i.e., removed all participants with missing outcome data from the analysis). Only 2% (2/83) of trial reports included a patient flow diagram, which would have clarified questions about missing data.

### Reporting of results

Almost three-quarters (72%, 60/83) of the trials presented outcome data as if they arose from a parallel group design. That is, instead of reporting the summary statistics (point estimate and precision estimate) of the within-individual difference with respect to an outcome, the investigators summed outcome measurements for a treatment from all participants across sequences [3,4]. For example, outcomes for treatment A were averaged across both sequence periods and outcomes for treatment B were averaged, and then the two averages were compared. An example of this type of inappropriate reporting of outcome data in crossover trials can be found in Table 2 from the publication of Konstas et al. [18]. This way of reporting ignored the paired nature of the design. A point estimate calculated this way is valid only when there are no missing data (i.e., the mean of differences equal the difference in means), but the estimate is less precise than the estimate calculated using the appropriate method. We also came across cases in which the reporting retained the paired nature of the design by examining the within-individual difference with respect to an outcome. However, the reporting was still incomplete because the treatment effect, the average of the outcome data for the two treatment sequences, was not reported (see Table 2 of Harasymowycz et al. [19]).

In our sample, almost all trials (94%, 78/83) reported a point estimate of treatment effect (Table 5), yet only one quarter of them (23%, 19/83) reported a standard deviation, a standard error, or a confidence interval on the estimated treatment effect that accounted for the paired nature of the design; one half of them (51%, 42/83) reported results of a hypothesis test for the treatment effect that accounted for the pairing (a t-statistic or a p-value from a paired sample t-test), and 5% (4/83) reported individual patient data.

**Table 5:** Reporting of results of included crossover trials (n = 83).

Study characteristics	Frequency	
	Number	%
**Included a patient flow diagram**	2	2
**Presented Individual patient/eye data**	4	5
**Presented results from the first period separately**	16	19
**Reporting of treatment effect estimates**		
Reported a point estimate (or one can be calculated)	78	94
Reported a precision estimate that accounted for the pairing^1^	19	23
Reported results of a hypothesis test that accounts for pairing^1^	42	51
**Presented data for each study group as if they were from a parallel group trial** ^**2**^	60	72

^1^See Fig 1 for details on the analysis of crossover trials.

^2^Undesirable way of reporting results from a crossover trial.

### Inclusion into a meta-analysis

A meta-analyst may decide to include or exclude crossover trials from a meta-analysis depending on the presumed assumptions made and approaches taken (Table 6). To include a study in a meta-analysis, one would need a point estimate (e.g., relative risk, mean difference) and associated precision of the point estimate (e.g., standard error, confidence interval). In our sample, only 60% (50/83) of trials reported these two data elements for inclusion in a meta-analysis without further assumptions and mathematical manipulations; that proportion decreased to 31% (26/83) if only crossover trials that used a washout period were considered appropriate for inclusion. Mathematical manipulation includes calculating precision of the point estimate using individual patient data when available or assuming a certain degree of correlation between the two measurements taken on the same individual [7]. Meta-analysts may also choose to use the data from the first period only; 19% (16/83) of trials in our sample would contribute data to the meta-analysis were we to follow this approach.

**Table 6:** Number of crossover trials that would be included in a meta-analysis, assuming inclusion based on different design and analysis characteristics (n = 83).

	Study characteristics	Frequency	
		Number	%
a:	Accounted for paired data nature of crossover trials in calculating the point estimate of treatment effect^1^	78	94
b.	Accounted for paired data nature of crossover trials in calculating the precision (e.g., standard error, confidence interval) of the point estimate of treatment effect^2^	50	60
c.	Presented individual patient data for all study groups	4	5
d.	Reported the results from the first period separately	16	19
e.	Used a washout period before crossing over to the next intervention	34	41
f.	a and b	50	60
g.	c or d or f	56	67
h.	g and e	26	31

^1^Or the point estimate is calculable, for example, based on the mean for each treatment sequence.

^2^Or the precision estimate is calculable, for example, based on a p-value.

## Discussion

Up-to-date systematic reviews and meta-analyses are an important way of summarizing the current status of information about treatment effectiveness and safety. In preparing for a systematic review of medical interventions for ocular hypertension and open-angle glaucoma, we found that a large number of eligible trials are crossover trials. For some disciplines, including ophthalmology subspecialties, crossover trials may be encountered quite often in the literature [8]. We believe that data from these trials are critical to presenting summary information; and not to include them would represent a waste of research information. As far as we know, the topic of inclusion of crossover trials into a meta-analysis has not been addressed with empirical data. For example, we struggled with which studies sufficiently minimized bias to merit inclusion as well as which results were based on paired analysis. We examined critical characteristics of the 83 eligible crossover trials and report on them here to facilitate further discussion. Our goal is to contribute to developing guidance and reporting standards for future investigators and systematic reviewers on areas of potential concern.

We found that the crossover design is attractive to investigators but easily can be misused. This has implications for our evidence base as a whole since the results may be of limited value to meta-analysts due to inappropriate analysis and inadequate reporting. In our sample, authors of only a few trials discussed the prerequisites of the crossover design. For example, there was limited information with regard to whether the underlying disease was likely to have a constant intensity during all treatment periods; the authors infrequently explored or discussed whether the effect of the treatment was likely to be restricted to the period in which it was applied (minimal carryover effect). Furthermore, some trials failed to accommodate the within-individual differences in the analysis, losing the statistical efficiency offered by the design. For a large proportion of the trials, the authors tabulated the results as if they arose from a parallel design. The precision estimates that had properly accounted for the paired nature of the design were often unavailable from the study reports; consequently, to include their findings in a meta-analysis would require further manipulation and assumptions.

We provide the following recommendations.

1. *Investigators who choose a crossover design should communicate the rationale to readers of the trial report for why such a design is suited to the condition and intervention in question*, *so that readers can gauge the suitability of the design and validity of the results*. Investigators may choose a crossover design over a parallel group design because of the potential savings in sample size, but sample size should not be the sole determinant. The advantages of the crossover design must be weighed carefully against its limitations, noted earlier. These considerations rely largely on investigator judgment since statistical testing has been shown to be unsatisfactory [9].
2. *Analysis for crossover trials must accommodate the paired nature of the design and the investigators should report explicitly the analytical methods in trial reports*. Crossover trials in which within-individual treatment comparisons are not made are of limited value because the potential savings in sample size (or gains in statistical efficiency) are not realized. Assuming that there is no carryover or period effect, one can take the difference of the measurement on intervention A minus measurement on intervention B *separately* for each participant (Fig 1). The mean and standard error of these differences serve as the building blocks for calculating the treatment effect and associated precision [1–3, 7]. Similar approaches could be applied to categorical data, and the recent statistical literature provides guidance [10–13]. Since a paired-sample analysis may not be familiar to everyone, involving a statistician in trial design and data analysis is likely to be beneficial.
3. *Investigators of crossover trials should report treatment effect estimates and precision estimates that properly accounted for the design*, *as well as other relevant data to facilitate understanding of any carryover effect and missing data*. We found that the reporting of treatment effects based on crossover trials is far from satisfactory. Because at least two measurements were made on the same individual, sometimes the authors reported twice the actual sample size in the results tables. Most notably, the precision estimates that accounted for the paired nature of the design were not available from a large proportion of trials, which reduced our confidence in an analysis overall. For quantitative results, we encourage researchers to report all elements indicated in Table 7. The cell-level means, standard deviations, and sample sizes in Table 7, although not directly reflecting treatment effects, are critical for the reader to understand the likelihood of carryover effect and period effect, as well as the amount of missing data. Reasons for missing data also should be reported transparently, for example, by using a patient flow diagram. Clinicaltrials.gov and other registers could adapt a similar schema for registering results from crossover trials.
4. *The baseline value(s) used to estimate treatment effect should be stated explicitly*. The use of change from baseline as an outcome metric in a crossover trial is common but not well supported. Despite its popularity in a parallel design, it is unlikely to be beneficial in a crossover design because the changes from baseline from the two treatment periods usually have a low correlation and the variance of the treatment effect estimates actually may be increased (less precise) [14]. Nevertheless, if baseline value(s) are used to calculate a change score from baseline, the baseline selected should be described clearly.

**Table 7:** Reporting of continuous outcomes from a two-treatment, two-period crossover trial.

	Treatment period		
Treatment sequence	1	2	Within-individual difference: A-B
**A then B**			
Mean (SD)	Y_A1_ (SD_A1_)	Y_B2_ (SD_B2_)	d1 (SD_d1_)
Sample size	n1	n2	n5
**B then A**			
Mean (SD)	Y_B1_ (SD_B1_)	Y_A2_ (SD_A2_)	d2 (SD_d2_)
Sample size	n3	n4	n6
**Treatment effect**			
Mean (SD)	-	-	d3 (SD_d3_) & CI
Sample size	-	-	n5 + n6
T-test for paired samples	-	-	P-value

SD: Standard deviation

CI: Confidence interval

n1 = n2 = n5 and n3 = n4 = n6 only when there are no missing data.

The cell level means are useful for estimating treatment by period interaction. Using the notations above, the treatment by period interaction = Y_A2_ + Y_B1_ –Y_B2 –_Y_A1_ [1,2]. The cell level sample sizes inform the extent of missing data and are useful for calculating standard errors.

Absence of reporting guidelines may help to explain the inadequate and sometime misleading reporting we observed in our sample. A CONSORT extension for reporting crossover trials is under development, which will be useful for journal editors as well as investigators. In addition to the above-mentioned issues specific to crossover trials, other elements described in the CONSORT statement for randomized controlled trials should also be carefully described [15]. Adequate reporting is also helpful for assessing the risk of bias of crossover trials [16].

In addition to disseminating possibly misleading information on the effects of interventions, poor reporting of crossover trials has negative downstream consequences. It precludes full use of crossover data in meta-analyses. Methods exist to transform and impute missing information so that crossover trials could be included [7,16]. For example, one can approximate the paired analysis by assuming a certain degree of correlation between two measurements taken on the same individual. When a carryover effect cannot be ruled out, one can use data collected from the first period in a meta-analysis (which might be biased) [9]. Yet as shown in this paper and demonstrated in the literature, most of these methods rely on assumptions and additional data manipulation, unnecessary steps when the reporting is accurate, complete, and appropriate to the design.

This multifaceted project is continuing. A future step will be to evaluate the impact of including different set of trials into the meta-analysis. We are in the process of publishing our main network meta-analysis on the comparative effectiveness of first-line topic medications for open angle glaucoma. We will be interested to see whether the relative effect estimates and rankings will change depending on whether trials meeting criteria f, g, or h on Table 6 are included in the network meta-analysis. Criterion h is the most stringent one and restricting analysis to this set of trials would be the least biased in theory.

Current practice of including crossover trials in a meta-analysis varies. Elbourne and colleagues examined 184 systematic reviews that mentioned including crossover trials. They found that 17% of them excluded crossover trials from the meta-analysis, about a half used data from the first period of the trial only, and a third included data from both periods as though a parallel group design had been used; only one review (1%) incorporated the paired data into the meta-analysis [7]. A more recent study by Lathryis and colleagues had similar findings: only 1/33 meta-analyses they examined stated that the paired data had been incorporated into the meta-analysis [17]. Thus, the crossover design is not well understood by most authors conducting systematic reviewers. Because the methods for including crossover trials into a meta-analysis may not be familiar to the usual systematic reviewer, we recommend working with a statistician. When the data from crossover trials cannot be incorporated fully into a systematic review and meta-analysis, full benefit of the trial is not realized.

In conclusion, the value of crossover trials to clinicians, their patients, and systematic reviewers depends on the appropriateness of the design, conduct, as well as the quality of reporting. There is pressing need for reporting guidelines for crossover trials. Guidance is needed if we are to incorporate crossover trial findings into meta-analyses.

## Supporting Information

S1 File Data abstraction form for crossover trials. (PDF)
