# Supplementary material for: Design, Analysis, and Reporting of Crossover Trials for Inclusion in a Meta-Analysis
Source: PLoS One. 2015 Aug 18;10(8):e0133023. doi: 10.1371/journal.pone.0133023 (PMC4540315; doi:10.1371/journal.pone.0133023)
Supplement: S1 File — (PDF) [file pone.0133023.s001.pdf]

PDF ID: ___ ___ ___ ___

Data Abstraction Form for Crossover Trials

Purpose:

This form is a paper version of an online form. The purpose of this form is to assess the design, analysis, and reporting of crossover trials on medical interventions for management of primary open angle glaucoma or ocular hypertension.

Completed by whom:

(At least) two independent data abstractors. Questions 32-41 must be completed by data abstractors with relevant statistical knowledge in the analysis of crossover trials.

1. Please verify that the PDF matches with the citation information.

(1) Yes

(2) No… 1a. What is the citation of your PDF? ____

2. Did the authors give any explanation for using a crossover design?

Yes	No	
(1)	(2)	a. Potential saving in resources (e.g., smaller sample size or fewer observations)
(1)	(2)	b. The condition is suitable for a crossover design (e.g., chronic disease)
(1)	(2)	c. The trial assessed patient preferences
(1)	(2)	d. As a way to permit within-patient comparisons
(1)	(2)	e. Others… 2f. If yes, please specify: ____
(1)	(2)	g. No explanations
(1)	(2)	h. Cannot tell

3. How many intervention strategies were studied altogether?

(1) 2

(2) 3

(3) More than 3… 3a. Please specify the number of intervention strategies: ____________________

4. Was the trial an AB/BA design?

The simplest cross-over trial is the two-treatment (A and B) two-period design, or the AB/BA design. In this type of study, there are two treatment periods and participants are randomized to receive either A followed by B or B followed by A.

(1) Yes

(2) No

(8) Not applicable

(9) Cannot tell

5. Was there a washout period before a participant crossed over to the next intervention?

Washout period is the interval of time considered necessary for a biological system to remove a foreign substance and to be free of its influence.

(1) Yes... 5a. Please specify the length of washout period, including time unit: ___________________

(2) There was no washout period AND no statement about why

(3) There was no washout period AND the authors stated why a washout period was not needed

(9) Cannot tell

6. Did any outcome measure reflect "a change score from baseline"?

(1) Yes

(2) No ***(SKIP to Question 8)***

(9) Cannot tell ***(SKIP to Question 8)***

7. <u>In the second period</u>, which values were used for calculating the "change score from baseline"?

Yes	No	
(1)	(2)	a. Values taken before the start of the first treatment
(1)	(2)	b. Values taken after the completion of the first treatment and before the start of the second treatment
(1)	(2)	c. Not reported/Cannot tell

8. Did the authors report a *pre-planned* sample size calculation?

An example of reporting a preplanned sample size calculation: "Before the study started, it was determined that a sample size of 20 patients was needed to achieve a 80% power."

(1) Yes… 8a. What was the power assumed? ____

(2) No

(9) Cannot tell

9. Did the authors report a *post-hoc* power calculation based on the available sample size (e.g., conditional power)?

An example of reporting a post-hoc power calculation based on the available sample size: "With 27 patients completing the study, the study provided a 60% power to detect an IOP difference of 1.5 mmHg."

(1) Yes… 9a. What was the power estimated? ____

(2) No

(9) Cannot tell

10. What statistical method(s) did the authors use to estimate treatment effects?

Yes	No	
(1)	(2)	a. T test
(1)	(2)	h. Paired t test*
(1)	(2)	b. Wilcoxon signed rank test
(1)	(2)	c. Analysis of variance (ANOVA)/Analysis of covariance (ANCOVA)
(1)	(2)	d. Multi-level model or mixed model method
(1)	(2)	e. Others… 10f. If yes, please specify: ____
(1)	(2)	g. Not reported/Cannot tell

*Item h was newly added in form version 2.0

11. Did the authors mention the concept of "period effect" anywhere in the article?

Period effect is a tendency affecting the crossover trial as a whole. It is a general tendency that even if the participants were given identical treatments in both periods, values in the second treatment period would be always higher (or always lower) than those in the first.

(1) Yes

(2) No ***(SKIP to Question 17)***

(9) Cannot tell

12. Did the authors perform any statistical test to assess the presence of a “period effect” (please see page 12 for descriptions)?

(1) Yes

(2) No

(9) Not reported/Cannot tell

13. Did the authors make an attempt to deal with “period effect” in the analysis?

(1) Yes

(2) No ***(SKIP to Question 15)***

(9) Not reported/Cannot tell

14. What did the authors say about dealing with “period effect” in the analysis?

Yes	No	
(1)	(2)	a. Performed CROS-based analysis (please see page 12 for descriptions)
(1)	(2)	b. Included “period” as a covariate in the statistical model
(1)	(2)	c. Used other approach(es) to deal with “period effect”… 14d. If yes, please specify: ________________________________
(1)	(2)	e. Not reported/Cannot tell

15. Did the authors say anything about “period effect” in the *Results* section?

(1) Yes

(2) No

(9) Cannot tell

16. Did the authors discuss “period effect” in the *Discussion* section?

(1) Yes

(2) No

(9) Cannot tell

17. Did the authors mention the concept of "carryover effect" anywhere in the article?

Carryover effect is the treatment effect from one period continues to be present during the following period. In AB/BA crossover trials, carryover effect and treatment by period interaction are not distinguishable.

(1) Yes

(2) No ***(SKIP to Question 23)***

(9) Cannot tell

18. Did the authors perform any statistical test to assess the presence of a "carryover effect" (please see page 12 for descriptions)?

(1) Yes

(2) No

(9) Not reported/Cannot tell

19. Did the authors make an attempt to deal with "carryover effect" in the analysis?

(1) Yes

(2) No ***(SKIP to Question 21)***

(9) Not reported/Cannot tell

20. What did the authors say about dealing with "carryover effect" in the analysis?

(1) Assumed no carryover effect in the analysis

(2) In AB/BA trials, the authors accounted for treatment by period interaction in the analysis

(3) Used some statistical approach to deal with "carryover effect"… 20a. Please specify: __________
____

(9) Not reported/Cannot tell

21. Did the authors say anything about "carryover effect" in the *Results* section?

(1) Yes

(2) No

(9) Cannot tell

22. Did the authors discuss "carryover effect" in the *Discussion* section?

(1) Yes

(2) No

(9) Cannot tell

23. Did the authors include a patient flow diagram?

Patient flow diagram is a figure that describes the progress through the phases of a clinical trial, i.e., enrolment, intervention allocation, follow-up, and data analysis.

(1) Yes

(2) No

(9) Cannot tell

24. Were individual patient/eye data presented for all study groups in a way that they can be used to calculate treatment effect?

(1) Yes

(2) No

(9) Cannot tell

Use the "primary analysis" in the article to answer Question 25-41.

"Primary analysis" is the analysis of primary outcome if primary outcome was specified. Otherwise, "primary analysis" is the analysis of the primary results (judged by the data abstractor).

25. What outcome of interest was used for the "primary analysis"?

(1) Intraocular pressure

(2) Visual field

(3) Optic nerve progression

(4) Visual acuity

(5) Others… 25a. Please specify: ____

26. What was randomized?

<u>Yes</u> <u>No</u>

(1) (2) a. When one eye eligible, participant/eye randomized

(1) (2) b. When two eyes eligible, only one eye per participant randomized (e.g., worse eye, left/right eye was randomized) and the fellow eye was not given any study intervention

(1) (2) c. When two eyes eligible, randomized two eyes independently

(1) (2) d. When two eyes eligible, randomized only the 1st eye and the fellow eye was given the different study intervention

(1) (2) e. When two eyes eligible, participant randomized (i.e., always randomized two eyes of a participant to the same intervention)

(1) (2) f. Others… 26g. If yes, please specify: ____

(1) (2) h. Not reported/Cannot tell

27. When both eyes from a participant were randomized, what was the unit for analysis?

(1) Left eye ***(SKIP to Question 29)***

(2) Right eye ***(SKIP to Question 29)***

(3) Average of two eyes ***(SKIP to Question 29)***

(4) The eye with the higher intraocular pressure ***(SKIP to Question 29)***

(5) The eye with the lower intraocular pressure ***(SKIP to Question 29)***

(6) One eye chosen at random ***(SKIP to Question 29)***

(7) Analyzed two eyes separately

(8) Cannot tell

(9) Not applicable ***(SKIP to Question 29)***

(10) Others… 27a. Please specify: ____

28. Did the authors say that they accounted for the paired-eye design in the analysis when estimating the treatment effect?

(1) Yes

(2) No

(8) Not applicable

(9) Cannot tell

29. How many patients/eyes were randomized?

<u>Yes</u> <u>No</u>

($_1$) ($_2$) a. Not reported

($_1$) ($_2$) b. # of eyes… 29c. If yes, please specify: ____

($_1$) ($_2$) d. # of patients… 29e. If yes, please specify: _____________________________________

30. Were there any missing data for the "primary analysis"?

Missing data can arise for the following reasons: participants were excluded from the study after randomization, lost to follow-up, participants withdrew from the study, missed a follow-up visit, or data collected were incomplete.

($_1$) Yes

($_2$) No ***(SKIP to the text before Question 32)***

($_9$) Cannot tell

31. What did the authors say about dealing with missing data in the context of a crossover design?

<u>Yes</u> <u>No</u>

($_1$) ($_2$) a. Used statistical methods to deal with missing data…31b. If yes, please specify the methods: ____

($_1$) ($_2$) c. Excluded patients/eyes with missing outcome data

($_1$) ($_2$) d. Not reported/Cannot tell

Questions 32-41 must be completed by data abstractors with relevant statistical knowledge in the analysis of crossover trials.

32. Which of the following periods did the authors use data from?

($_1$) One period only ***(SKIP to Question 40)***

($_2$) More than one period

($_9$) Cannot tell

33. Did the authors say that they accounted for the paired data nature of crossover trials in the analysis when estimating the treatment effect?

($_1$) Yes… 33a. Please specify what they said: ____

($_2$) No

($_9$) Cannot tell

34. In your opinion, did the authors account for the paired data nature of crossover trials in the analysis when estimating the treatment effect?

(1) Yes

(2) No

(9) Cannot tell

35. Were the only available point estimates for outcomes (e.g., mean IOP) presented separately for each study group as if the data were from a parallel group trial?

(1) Yes

(2) No

(9) Cannot tell

36. In your opinion, did the authors report point estimates of treatment effect that accounted for the crossover design (or the point estimates are calculable)?

(1) Yes

(2) No

(9) Cannot tell

37. In your opinion, did the authors report the precision (e.g., SD, SE or CI) of the point estimates of treatment effect that accounted for the crossover design (or the precision is calculable)?

(1) Yes

(2) No

(9) Cannot tell

38. In your opinion, did the authors report the results of a hypothesis testing (e.g., exact p-value of a paired t test) for the point estimates of treatment effect that accounted for the crossover design (or the p-value is calculable)?

If the authors reported a range, for example p-value <0.05, please select "No."

(1) Yes

(2) No

(9) Cannot tell

39. Did the authors report the results from the first period separately?

($_1$) Yes

($_2$) No

($_9$) Cannot tell

40. In your opinion, are there any quantitative data on treatment effect that can be included in a meta-analysis (regardless of whether the precision estimates accounted for the paired-design)?

For example, when a trial reported results from the 1st period or when a trial was analyzed as if it was a parallel group trial, we can still use the quantitative data for meta-analysis, albeit the precision estimates from the trial are larger than they should be under both scenarios.

($_1$) Yes

($_2$) No

($_9$) Cannot tell

41. In your opinion, will you feel comfortable to include the quantitative data from this trial in a meta-analysis?

($_1$) Yes

($_2$) No… 41a. Please justify your answer briefly: ____

($_9$) Cannot tell

42. Please specify what data need to be abstracted for meta-analysis (e.g., effect estimates and standard errors for intraocular pressure in Table 2). Write “None” if your answer to Question 41 is “No” or “Cannot tell”.

____

____

43. Any comments you may have (Type “None” if you don't have any comments):

____

____

Administrative Information

44. Date form completed (MM/DD/YYYY): ___ ___ / ___ ___ / ___ ___ ___ ___

45. Initials of the data abstractor: ___ ___

46. Name of the data abstractor: ____

First Last

PDF ID: ___ ___ ___ ___

BASIC MODEL FOR CROSSOVER TRIALS

$\mu - expected\ result\ after\ treatment\ A$

$\delta - additional\ benefit\ of\ treatment\ B$

	Period 1	Period 2
Group 1 (A→B)	$E\ \ Y_{11}\ \ = \mu$	$E\ \ Y_{12}\ \ = \mu + \delta$
Group 2 (B→A)	$E\ \ Y_{21}\ \ = \mu + \delta$	$E\ \ Y_{22}\ \ = \mu$

MODEL WITH PERIOD EFFECT

$\mu - expected\ result\ after\ treatment\ A$

$\pi - period\ effect\ of\ period\ 2$

$\delta - additional\ benefit\ of\ treatment\ B$

	Period 1	Period 2
Group 1 (A→B)	$E\ \ Y_{11}\ \ = \mu$	$E\ \ Y_{12}\ \ = \mu + \pi + \delta$
Group 2 (B→A)	$E\ \ Y_{21}\ \ = \mu + \delta$	$E\ \ Y_{22}\ \ = \mu + \pi$

Estimation of treatment effect, assuming no carryover effect

$CROS = [(Y_{12} - Y_{11}) +\ \ Y_{21} - Y_{22}\ \ ]/2 = [(Y_{12} - Y_{11}) -\ \ Y_{22} - Y_{21}\ \ ]/2$

$E\ \ CROS\ \ =\ \ \pi + \delta - \pi + \delta\ \ /2 = \delta$

MODEL WITH PERIOD EFFECT AND CARRYOVER EFFECT

$\mu - expected\ result\ after\ treatment\ A$

$\pi - period\ effect\ of\ period\ 2$

$\delta - additional\ benefit\ of\ treatment\ B$

$\lambda_A\ and\ \lambda_B - carryover\ effect\ of\ treatment\ A\ and\ B, respectively$

$\lambda = \lambda_A - \lambda_B - difference\ of\ carryover\ effects$

	Period 1	Period 2
Group 1 (A→B)	$E\ \ Y_{11}\ \ = \mu$	$E\ \ Y_{12}\ \ = \mu + \pi + \delta + \lambda_A$
Group 2 (B→A)	$E\ \ Y_{21}\ \ = \mu + \delta$	$E\ \ Y_{22}\ \ = \mu + \pi + \lambda_B$

$CROS = [(Y_{12} - Y_{11}) -\ \ Y_{22} - Y_{21}\ \ ]/2$

$E\ \ CROS\ \ = \delta - \lambda/2$

$SEQ = (Y_{21} + Y_{22}) -\ \ Y_{11} + Y_{12}$

$E\ \ SEQ\ \ = \lambda$
